# In vivo polarisation sensitive optical coherence tomography for fibrosis assessment in interstitial lung disease: a prospective, exploratory, observational study

**DOI:** 10.1136/bmjresp-2023-001628

**Published:** 2023-08-08

**Authors:** Margherita Vaselli, Kirsten Kalverda-Mooij, Erik Thunnissen, Michael W T Tanck, Onno M Mets, Inge A H van den Berk, Jouke T Annema, Peter I Bonta, Johannes F de Boer

**Affiliations:** 1 Department of Physics and Astronomy, Vrije Universiteit Amsterdam, Amsterdam, The Netherlands; 2 Respiratory Medicine, Amsterdam University Medical Centres, Amsterdam, The Netherlands; 3 Department of Pathology, Amsterdam University Medical Centra, Amsterdam, The Netherlands; 4 Department of Epidemiology and Data Science, University of Amsterdam, Amsterdam, The Netherlands; 5 Department of Radiology and Nuclear Medicine, University of Amsterdam, Amsterdam, The Netherlands; 6 Department of Radiology and Nuclear Medicine, Amsterdam UMC Location AMC, Amsterdam, The Netherlands; 7 Respiratory Medicine, Amsterdam UMC - Locatie AMC, Amsterdam, The Netherlands; 8 Amsterdam UMC - Locatie AMC, Amsterdam, The Netherlands

**Keywords:** Interstitial Fibrosis, Bronchoscopy, Imaging/CT MRI etc

## Abstract

**Introduction:**

Endobronchial polarisation sensitive optical coherence tomography (EB-PS-OCT) is a bronchoscopic imaging technique exceeding resolution of high-resolution CT (HRCT) by 50-fold. It detects collagen birefringence, enabling identification and quantification of fibrosis.

**Study aim:**

To assess pulmonary fibrosis in interstitial lung diseases (ILD) patients with in vivo EB-PS-OCT using histology as reference standard.

**Primary objective:**

Visualisation and quantification of pulmonary fibrosis by EB-PS-OCT.

**Secondary objectives:**

Comparison of EB-PS-OCT and HRCT detected fibrosis with histology, identification of ILD histological features in EB-PS-OCT images and comparison of ex vivo PS-OCT results with histology.

**Methods:**

Observational prospective exploratory study. Patients with ILD scheduled for transbronchial cryobiopsy or surgical lung biopsy underwent in vivo EB-PS-OCT imaging prior to tissue acquisition. Asthma patients were included as non-fibrotic controls. Per imaged lung segment, fibrosis was automatically quantified assessing the birefringent area in EB-PS-OCT images. Fibrotic extent in corresponding HRCT areas and biopsies were compared with EB-PS-OCT detected fibrosis. Microscopic ILD features were identified on EB-PS-OCT images and matched with biopsies from the same segment.

**Results:**

19 patients were included (16 ILD; 3 asthma). In 49 in vivo imaged airway segments the parenchymal birefringent area was successfully quantified and ranged from 2.54% (no to minimal fibrosis) to 21.01% (extensive fibrosis). Increased EB-PS-OCT detected birefringent area corresponded to increased histologically confirmed fibrosis, with better predictive value than HRCT. Microscopic ILD features were identified on both in vivo and ex vivo PS-OCT images.

**Conclusions:**

EB-PS-OCT enables pulmonary fibrosis quantification, thereby has potential to serve as an add-on bronchoscopic imaging technique to diagnose and detect (early) fibrosis in ILD.

**WHAT IS ALREADY KNOWN ON THIS TOPIC**

- In patients with an interstitial lung disease (ILD), accurate diagnosis and early recognition of progressive pulmonary fibrosis is challenging but important for classification, treatment and prognosis. Endobronchial optical coherence tomography (OCT) is a minimally invasive, bronchoscopic, imaging modality to generate volumetric images of the lung parenchyma with microscopic resolution. Polarisation sensitive OCT (PS-OCT) is an extension of conventional OCT that adds birefringence detection. With fibrosis being highly birefringent, PS-OCT may enable fibrosis detection in the lung parenchyma.

**WHAT THIS STUDY ADDS**

- In this study, we show that in vivo endobronchial PS-OCT can automatically quantify fibrosis and detect ILD features in patients with ILD by minimally invasive bronchoscopy.

**HOW THIS STUDY MIGHT AFFECT RESEARCH, PRACTICE OR POLICY**

- Endobronchial PS-OCT has potential to serve as an add-on bronchoscopic imaging technique to diagnose and assess (progressive) fibrosis in patients with ILD.

## Introduction

Interstitial lung diseases (ILDs) include a large group of different diseases, characterised by inflammation and pulmonary fibrosis.^1^ Treatment consists of anti-inflammatory and/or antifibrotic medication. ILD diagnosis and identification of patients with ILD with a (progressive) fibrotic phenotype is challenging, but of key importance for treatment decisions and prognosis.^2–4^

For ILD diagnosis, no single diagnostic test exists. Guidelines recommend evaluating all available diagnostic information during a multidisciplinary team (MDT) discussion to establish a high confident diagnosis.^5^ To identify the fibrotic phenotype, pulmonary function testing and high-resolution CT (HRCT) are used as diagnostic parameters. However, changes in pulmonary function tests can be non-specific and HRCT has limitations in fibrosis detection and in differentiating fibrotic from inflammatory progression. Indeed, a weak correlation exists between fibrotic extent as detected by HRCT and prognosis.^6 7^ For disease pattern identification, lung biopsy is considered to be the reference standard, but is associated with morbidity and in rare case mortality. Lung tissue acquisition is, therefore, only performed in highly selected patients with ILD and is not appropriate for longitudinal measurements to evaluate fibrotic progression. Thus, there is an unmet need in fibrotic ILD diagnostics and ILD progression detection.

Conventional optical coherence tomography (OCT) is an imaging technique that uses near-infrared light to provide structural images of tissue with a resolution of ~10 µm.^8^ Endobronchial OCT (EB-OCT) refers to a technique in which a thin flexible OCT probe is advanced through the working channel of the bronchoscope to the periphery of the lung. The procedure is minimally invasive, can be performed under conscious sedation and can easily be combined with the standard diagnostic techniques including broncho alveolar lavage. In the last decade, EB-OCT emerged as a bronchoscopic imaging modality to generate cross-sectional images of the airways and lung parenchyma. Several studies reported EB-OCT in obstructive lung diseases,^9–12^ detection of malignancies^13–15^ and ILDs.^16 17^ Conventional EB-OCT provides volumetric reconstructions of lung tissue with microscopic resolution, but has limitations in fibrotic tissue-specific contrast.^18^ Polarisation sensitive OCT (PS-OCT) is an extension of conventional OCT that adds tissue-specific contrast by assessing presence of birefringence. Birefringence is an optical property exhibited by collagen in fibrous tissues including muscle, connective tissue and nerve fibres.^19^ As pulmonary fibrosis is characterised by an increase in collagen, PS-OCT can visualise pulmonary fibrosis. To date, the potential of EB-PS-OCT in pulmonary medicine has been demonstrated in vivo in a canine model^20^ and in asthma patients^21 22^ to detect airway smooth muscle reduction after bronchial thermoplasty. Recently, Nandy *et al* performed EB-PS-OCT in patients with ILD and manually quantified birefringence across tissue types including destructive and interstitial fibrosis.^23^

In this study, we use in vivo EB-PS-OCT to automatically quantify the area of birefringent tissue to assess fibrotic content in patients with ILD. For the first time, we compare the ability of EB-PS-OCT and HRCT for fibrosis detection and quantification using histopathology as reference standard. Additionally, we retrieve the orientation of birefringent tissues fibres, which provides a valuable tool to distinguish fibrous tissue from other birefringent tissues such as airway smooth muscle and perichondrium.

## Methods

### Study design and patient enrolment

This imaging study is a substudy of a randomised controlled trial comparing transbronchial cryobiopsy (TBCB), followed by surgical lung biopsy (SLB) and immediate SLB for ILD (COLD-study NTR NL7634). In this study, we aim to assess pulmonary fibrosis using in vivo EB-PS-OCT. The primary objective is to visualise and automatically quantify pulmonary fibrosis with in vivo EB-PS-OCT. Secondary study objectives are as follows: (1) compare EB-PS-OCT detected fibrosis with the extent of fibrosis as detected by HRCT and histology and (2) the identification of ILD hallmarks in PS-OCT images acquired both in vivo and ex vivo.

Considering this is an exploratory study no formal sample size calculation was performed.

Each subject provided separate written informed consent for this substudy. Patients with an undiagnosed ILD scheduled for lung biopsy in Amsterdam UMC were eligible for study participation. A full list of inclusion and exclusion criteria for this substudy are provided in online supplemental e-Table 1. For non-fibrotic controls, we used EB-PS-OCT measurements of asthma patients included in the EB-PS-OCT asthma study.^21^

10.1136/bmjresp-2023-001628.supp1 Supplementary data

### Patient and public involvement

For this exploratory study, patients were not involved in the design, conduct, reporting or dissemination plans.

### EB-PS-OCT imaging and fibrosis quantification

Prior to cryobiopsy or SLB, EB-PS-OCT measurements were performed by pulmonologists experienced in EB imaging techniques (PB, JTA and KK-M). Regions of interest (airway segments) for EB-PS-OCT imaging were identified by the pulmonologists based on HRCT scan and planned biopsy location. Patients were deeply sedated, remaining spontaneous breathing and intubated (cryobiopsy procedure) or under full anaesthesia (SLB procedure). In each patient, three different segments were imaged with EB-PS-OCT, and each segment was imaged at least twice. Volumetric EB-PS-OCT images were acquired by advancing the EB-PS-OCT probe through the working channel of the bronchoscope to the periphery of each lung segment until pleural resistance (online supplemental e-figure 1). The probe was then manually pulled back over a length of ~5–6 cm and three-dimensional volumetric images were acquired.

EB-PS-OCT measurements were performed through a distal scanning catheter, with a diameter of 1.35 mm, rotating at 52 rotations/s and protected by a medical grade pebax sheath.^24^ The probe was connected to a PS-OCT system, previously described,^24^ with an axial and lateral tissue resolution of 12 µm and 13 µm, respectively (refractive index n=1.4), and an imaging depth of 1.4 mm. Postprocessing analysis of the acquired EB-PS-OCT raw-data generated conventional OCT intensity images and EB-PS-OCT (optic axis orientation) images, in which birefringent structures and their fibres orientation were visualised and segmented.

For each airway segment imaged, fibrosis was automatically quantified in postprocessing as mean percentage of birefringence area in EB-PS-OCT cross-sectional images of the parenchymal (distal) part of all pullbacks acquired in that segment. EB-PS-OCT images of the proximal part of each pullback imaging the airway were not included in the fibrosis quantification given the prevalence of other birefringent structures in the proximal airway (eg, airway smooth muscle, perichondrium). Detailed information on EB-PS-OCT data processing and fibrosis quantification is provided in online supplemental e-appendix 1.

### Radiological and histological evaluation

HRCT scans were evaluated by two ILD-specialised radiologists (OMM and PB) (blinded for EB-PS-OCT results and to each other). Per EB-PS-OCT imaged and biopsied segment, a fibrosis score on HRCT was determined ranging from 0 to 3 (0=no fibrosis, 1=mild fibrosis, 2=moderate fibrosis, 3=severe fibrosis). In a consensus meeting, inconsistencies were discussed, and a final score was obtained. A lung specialised pathologist (ET), blinded for clinical information including HRCT and EB-PS-OCT results, evaluated the acquired biopsies for the presence of fibrosis on a 0–3 scale. Following a 6-week wash-out period, histological evaluation was repeated evaluating the biopsies for the presence of fibrosis in a different order, and blinded for previous evaluation. When a segment was biopsied twice, the overall score included both biopsies. In case of inconsistent scores, a third evaluation was performed 2 weeks after the last histological evaluation, and a final score was obtained. Criteria for the categorical fibrosis scoring system for HRCT and histology are provided in online supplemental e-appendix 2.

### Histology matching

Ex vivo PS-OCT measurements of the resected biopsies (11 SLB from 4 patients) were performed immediately after tissue extraction on the unprocessed specimen by pulling back the catheter while pressing it to the biopsy surface. All the biopsies were processed according to local standard practice. The histology sections were cut perpendicularly to the probe pullback direction. Ex vivo EB-PS-OCT images were compared with histology by the OCT expert (MV) and the pathologist (ET) for one-to-one matching of PS-OCT cross-sectional images with the histology sections obtained.

### Statistical analysis

Statistical analysis to compare accuracy of fibrosis classification between EB-PS-OCT versus histology and HRCT versus histology was performed on segments from which biopsies were obtained. Asthmatic controls with normal lung parenchyma on HRCT and normal diffusion capacity on pulmonary function test served as control.

Ordinal logistic regression was chosen to model the relationship between the response ordinal variable (histological fibrosis score) and the explanatory variables (EB-PS-OCT and HRCT detected fibrosis).

To correct for multiple segments within the same patient, the ordinal logistic regression was used with a random patient effect (cumulative link mixed model). The performance of the two models (EB-PS-OCT and histology vs HRCT and histology) was compared using the area under the receiver operating characteristic curve (generalised AUROC or c-index, specific measure for ordinal outcomes^25^) and the overall accuracy (calculated using a confusion matrix). Weighted kappa was used to evaluate inter and intra observer variability between radiologists evaluating HRCT scans and the pathologist evaluating histology slides. All analyses were carried out in R (V.4.1.3).

## Results

### Study cohort

Between January 2020 and July 2021, 16 patients with ILD who underwent lung biopsy were included (12 TBCB, 3 SLB and 1 both). Moreover, three asthma patients from a historical cohort were included as a non-fibrotic control group. These three patients were imaged with EB-PS-OCT 6 months following bronchial thermoplasty to assess airway smooth muscle thickness in the proximal airways, but EB-PS-OCT pullbacks imaged both airways and lung parenchyma.^21^ Baseline characteristics and final MDT-ILD diagnoses are represented in table 1. EB-PS-OCT imaging added ~5–10 min to the biopsy procedure without any procedure-related adverse events.

**Table 1:** Baseline characteristics and final diagnosis of patients with ILD

	ILD patients n=16	Asthma patients n=3
Gender		
M	11 (69)	1 (33)
F	5 (31)	2 (67)
Age	64±8	46±15
BMI	28±3	31±5
Smoking status		
Never smokers	2 (12.5)	3 (100)
Ex-smokers	12 (75)	
Current smoker	2 (12.5)	
Packyears (current and ex-smokers)	21±11	
Pulmonary function test		
FVC, % of predicted value	86±14	94±2
DLCOc, % of predicted value	58±15	92±8
Final ILD diagnosis		
cHP	7 (44)	
IPF	4 (25)	
srILD	1 (6)	
IPAF-fNSIP	1 (6)	
Idiopathic fNSIP	1 (6)	
Sarcoidosis	1 (6)	
Unclassifiable ILD	1 (6)	
Non-fibrotic asthma controls		3 (100)

Data are shown as N (%) or mean (±SD).

BMI, body mass index; cHP, chronic hypersensitivity pneumonitis; DLCOc, diffusing capacity of the lung for carbon monoxide corrected for haemoglobin; F, female; fNSIP, fibrotic non-specific idiopathic pneumonitis; FVC, forced vital capacity; ILD, interstitial lung disease; IPAF, interstitial pneumonia with autoimmune features; IPF, idiopathic pulmonary fibrosis; M, male; srILD, smoking-related ILD.

Out of 19 included patients, 17 (14 ILD and 3 asthma patients) underwent in vivo EB-PS-OCT imaging of 3 different lung segments. Each segment was imaged at least 2 times, resulting in a total of 128 in vivo pullbacks (400–800 frames per pullback). Two SLB ILD patients in which in vivo EB-PS-OCT was not performed for logistical reasons, were only included for ex vivo PS-OCT imaging.

In vivo EB-PS-OCT imaging was performed in a total of 55 individual segments. Six segments were excluded: n=5 segments, due to the pullback being too proximal and not containing alveolar tissue, and n=1, due to probe malfunction. Since in one patient all pullbacks were too proximal, this led to the exclusion of this patient.

**Figure 1:**
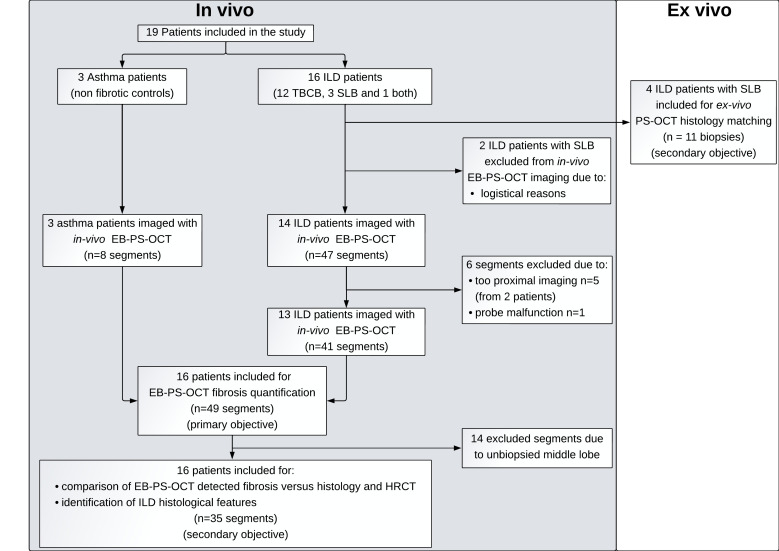
Flow chart of the EB-PS-OCT imaging study. Flow chart shows how patients relate to EB-PS-OCT objectives allocation both in vivo and ex vivo. Nineteen patients were included in the study (3 asthma and 16 ILD). Sixteen patients with ILD underwent lung biopsy: 12 TBCB, 3 SLB and 1 patient underwent both. Only SLB was included for ex vivo analysis. Two patients with ILD undergoing SLB were excluded from in vivo EB-PS-OCT for logistical reasons and were only included for ex vivo PS-OCT imaging. A total of 55 segments were imaged in vivo (8 from 3 asthma controls and 47 from 14 patients with ILD). After exclusion of segments in which no peripheral lung parenchyma was visualised (n=5, from 2 patients) or unable to be analysed due to probe malfunction (n=1), 49 segments were included for primary objective analysis (8 from 3 asthma patients and 41 from patients with ILD). For secondary objectives, only segments with corresponding histology were included (35 segments from 16 patients). EB-PS-OCT, endobronchial polarisation sensitive optical coherence tomography; HRCT, high-resolution CT; ILD, interstitial lung disease; SLB, surgical lung biopsy; TBCB, transbronchial cryobiopsy;.

In 49 in vivo imaged lung segments from 16 patients (13 ILD and 3 asthma patients) fibrosis was quantified based on EB-PS-OCT detected birefringent area (figure 1, primary objective: visualisation and quantification of pulmonary fibrosis by EB-PS-OCT). For secondary objectives, in total 35 segments were analysed: 8 segments from asthma patients and 27 from patients with ILD as only those segments for which corresponding biopsies were available were included (in 24 segments TBCB’s were acquired (n=47 cryobiopsies) and in 3 segments SLB’s were acquired (n=3 surgical biopsies)) (figure 1).

### EB-PS-OCT fibrosis quantification

In 49 segments, parenchymal fibrosis was quantified based on in vivo EB-PS-OCT detected birefringence. Birefringence from the peripheral lung parenchyma ranged from 2.5% in patients without fibrosis up to 21.0% in a patient with extensive fibrosis. In non-fibrotic asthma controls, birefringence ranged from 3.3% to 6.8%.

In line with our previous study,^26^ pullbacks acquired in the same segment showed good reproducibility of the EB-PS-OCT detected birefringence content (online supplemental e-Figure 2).

In patients with ILD, 63% (n=60 pullbacks) of EB-PS-OCT imaging was performed in the lower lobes, 4% (n=4 pullbacks) was performed in the upper lobes and 33% of the pullbacks (n=31) were obtained from the middle lobe. The birefringence extent detected by EB-PS-OCT in the lower lobe and in the middle lobe of each patient were compared. Lower lobes consistently showed more birefringence than the middle lobes, especially in idiopathic pulmonary fibrosis (IPF) and chronic hypersensitivity pneumonitis (cHP) patients (online supplemental e-Figure 3).

### EB-PS-OCT detected fibrosis versus HR-CT detected fibrosis compared with histology

**Figure 2:**
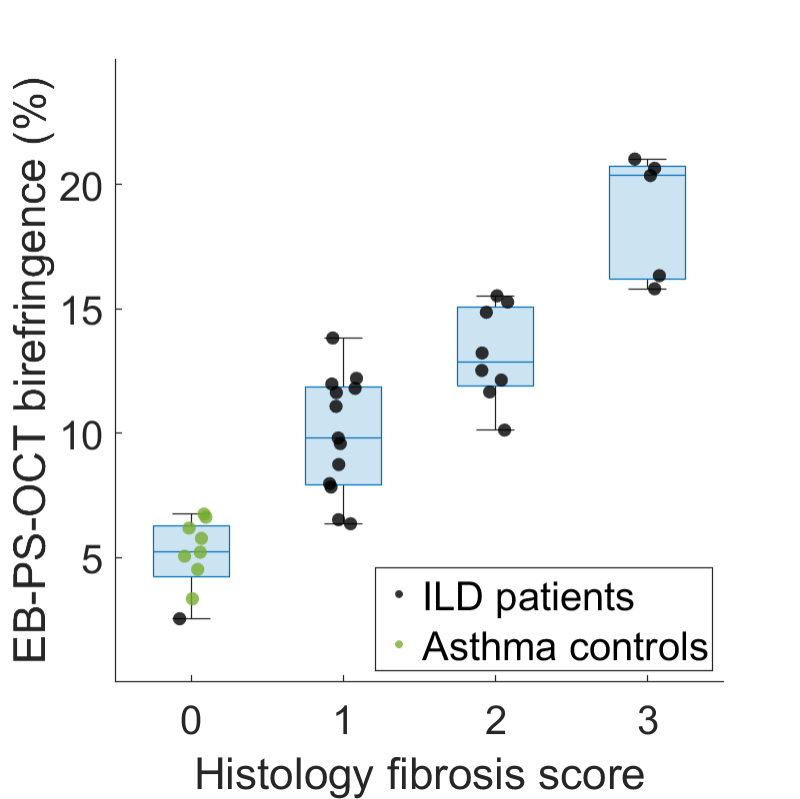
Quantitative measurement of birefringence % as detected by EB-PS-OCT (y-axis) compared with the histology fibrosis score (x-axis). Comparing the continuous data from EB-PS-OCT detected birefringence to the histology-based fibrosis score, higher EB-PS-OCT birefringence % corresponded to higher histological fibrosis scores. Each dot represents an EB-PS-OCT imaged and biopsied segment (n=35) from patients with ILD (black dots) and asthma controls (green dots). Birefringence from the peripheral lung parenchyma ranged from 2.5% in a patient with non-fibrotic ILD up to 21.0% in a patient with IPF. EB-PS-OCT, endobronchial polarisation sensitive optical coherence tomography; ILD, interstitial lung diseases; IPF, idiopathic pulmonary fibrosis.

Comparing the continuous data from EB-PS-OCT detected birefringence to the histology-based fibrosis score (n=8 Asthma, n=27 ILD), higher EB-PS-OCT birefringence values corresponded to higher histological fibrosis scores (figure 2). Lowest fibrosis scores were obtained from the asthma controls and a non-fibrotic ILD patient, and highest scores originate from an IPF and fibrotic cHP patient.

Current diagnostic tools including pulmonary function tests and HRCT have significant limitations in detection of fibrosis and are considered insufficient to detect subtle (progression of) fibrosis. To determine if EB-PS-OCT has added value over HRCT we compared EB-PS-OCT performance with HRCT to predict the fibrotic extent in histology. As histology was used as a reference standard, we included only the imaged segments for which histology was obtained (figure 1, secondary objective). Both HRCT and biopsies were analysed for extent of fibrosis by independent and blinded radiologists (Cohen’s kappa for interobserver agreement: 0.67 (95% CI 0.54 to 0.83) weighted kappa: 0.75) and a pathologist (Cohen’s kappa for intraobserver agreement: 0.88 (95% CI 0.76 to 0.99); weighted kappa of 0.91), respectively.

Based on an ordinal regression model, EB-PS-OCT shows higher discriminating performance than HRCT in predicting the histology fibrosis classification (AUROC (95% CI)) 0.96 (0.92 to 1.00) vs 0.83 (0.73 to 0.93) and overall accuracy (95% CI) 0.77 (0.60 to 0.90) vs 0.60 (0.42 to 0.76).

### In vivo EB-PS-OCT identified ILD hallmarks

**Figure 3:**
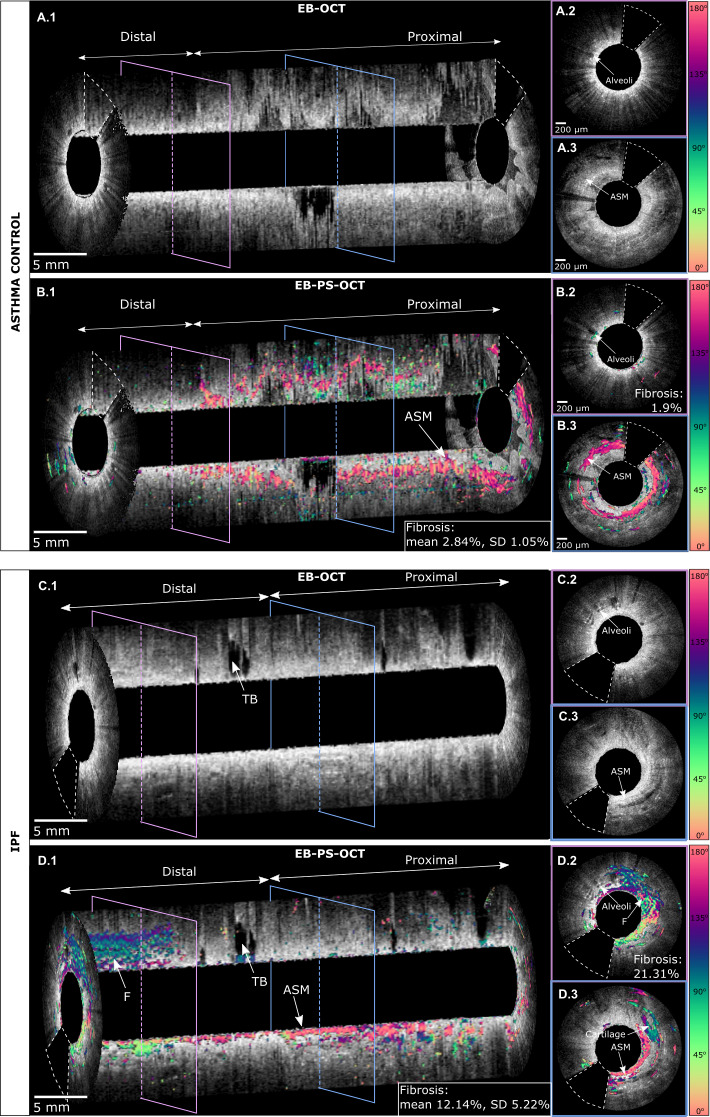
Conventional EB-OCT and EB-PS-OCT images of right lower lobe from an asthma patient (A, B), respectively and from an IPF patient (C, D), respectively. In the asthma patient, conventional EB-OCT visualises lattice-like signal-void areas recognised as normal alveoli in the lung parenchyma (A1) and in the cross-sectional image from the distal part of the pullback (A2). Cross-sectional image from the proximal part of the pullback shows airway smooth muscle layer structure (A3). The additional use of polarisation properties of light to the conventional EB-OCT image results in the EB-*PS*-OCT pullback image, where birefringent structures are visible in the different colours (B1). Each colour indicates a different orientation of the tissue optic axis (right sided colour scale). The absence of birefringence in the distal airway is shown by the absence of colours in the cross-sectional image of the distal part of the EB-PS-OCT pullback (B2). In the proximal part of the pullback a thick birefringent layer (in pink) indicates the presence of an airway smooth muscle layer (B3). In the IPF patient, conventional EB-OCT pullback (C1) shows the presence of a dilated, signal-void area connected to the main airway compatible with a traction bronchiectasis. The two cross-sectional images visualise alveolar tissue in the distal part of the pullback (C2) and in the proximal part of the pullback hypodense tissue representing airway smooth muscle (C3). Additionally, EB-PS-OCT enables the visualisation of regions of dense subpleural fibrosis indicated by the presence of highly birefringent areas with random optic axis orientation in the distal end of the pullback (D1) and in a cross-sectional image from the same area (D2). A cross-sectional image from the proximal part of the pullback (D3) shows the presence of airway smooth muscle (in pink) and of a cartilage structure surrounded by a birefringent perichondrium layer (in green). Optic axis orientation images have been calibrated using the birefringent catheter sheath; this enabled to determine that birefringent structures in pink are circumferentially oriented, while birefringent structures in green are longitudinally oriented. The white dotted lines in OCT and PS-OCT images delineate the tissue area not optically accessible because of the presence of the wires feeding current to the motor. ASM, airway smooth muscle; EB-PS-OCT, endobronchial polarisation sensitive optical coherence tomography; F, fibrosis; IPF, idiopathic pulmonary fibrosis; TB, traction bronchiectasis.

**Figure 4:**
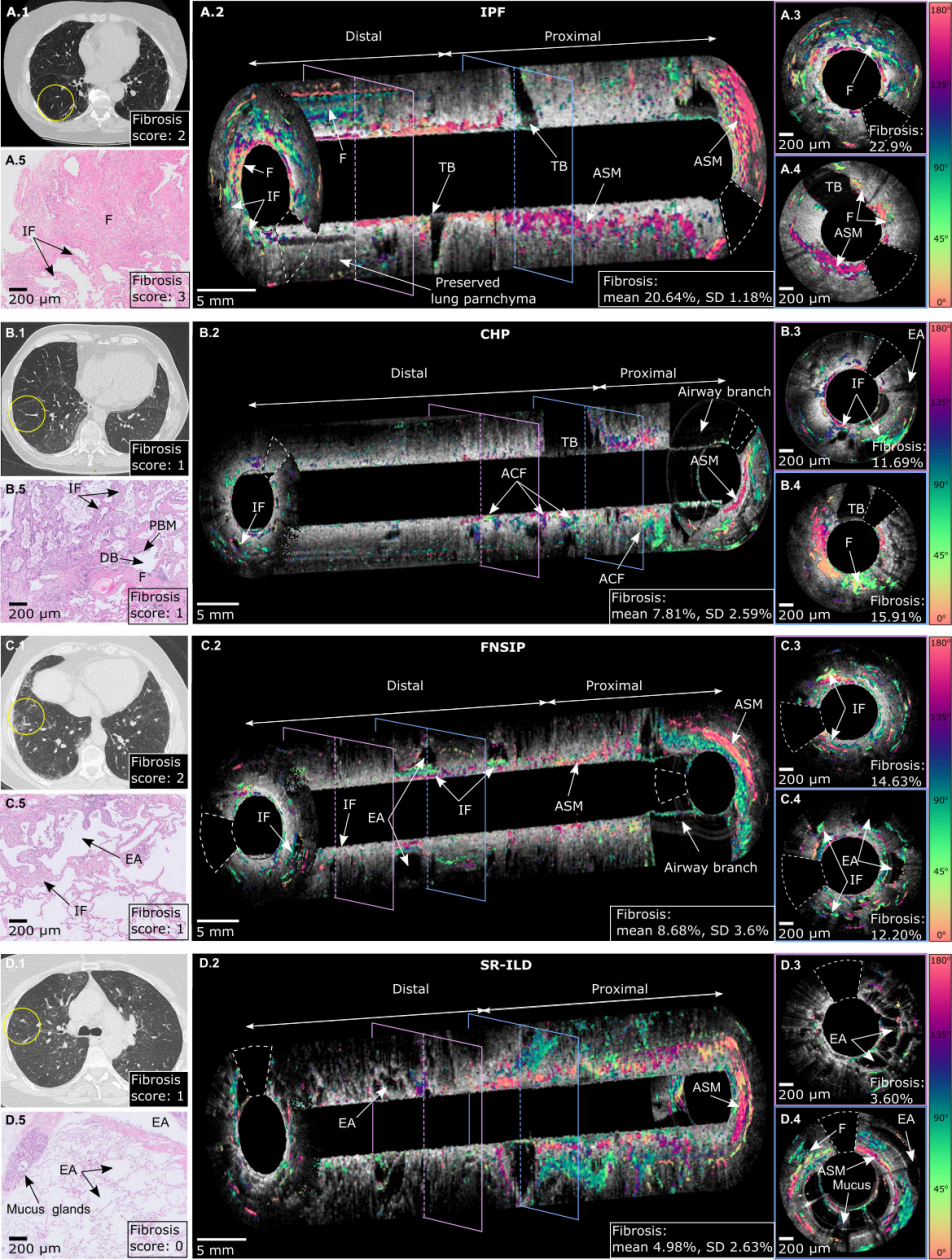
High-resolution CT (HRCT), H&E-stained histological section images and EB-PS-OCT images from patients diagnosed with IPF (A), cHP (B), fNSIP (C) and SR-ILD (D). Per pullback distal (parenchymal) part and proximal (airway) part are differentiated. For the evaluation of the amount of fibrosis in the lung parenchyma, only the distal part was analysed, as in the proximal part the amount of birefringence is higher due to other structures (eg, airway smooth muscle and cartilage). HRCT section indicates the segment where the EB-PS-OCT pullback was obtained (yellow circle) and the radiological fibrosis score (A1, B1, C1, D1). (A) IPF: regions of interstitial fibrosis and dense fibrosis alternate with regions of preserved lung parenchyma (A3). Traction bronchiectasis and airway smooth muscle are visible. The overall fibrosis of the distal area of the pullback is 20.64%. (A2). Proximal cross-sectional image shows little fibrosis and airway smooth muscle (A5). H&E-stained histology slide from the biopsied segment shows combination of dense fibrosis and interstitial fibrosis. (A5). (B) cHP: Interstitial fibrosis is noted subpleurally, most fibrotic changes are in the proximal part of the distal area, indicating airway centred fibrosis (B2). Overall fibrosis of the lung parenchyma was 7.81%. In areas of interstitial fibrosis and airway centred fibrosis enlarged alveolar spaces and traction bronchiectasis are visible (B3 and B4). Corresponding H&E histology shows interstitial fibrosis, peribronchial metaplasia and distal bronchiole (B5). (C) NSIP throughout the entire distal part of the pullback interstitial fibrosis is identified in a homogeneous pattern (C2). In areas of fibrosis enlarged alveolar spaces are visible (C2 and C4). Airway smooth muscle is identified, in the proximal part of the pullback (C2). Overall fibrosis in the distal part of the pullback was 8.68%. H&E histology shows mild fibrotic changes consisting of interstitial fibrosis (C5). (D) srILD: hardly any fibrosis visible in the parenchymal part of the pullback (D2) (EB-PS-OCT detected fibrosis in the distal part of the pullback is 4.98%) (D2). The extensive amount of birefringence in the proximal part of the pullback is compatible with airway remodelling (D2). Enlarged alveolar spaces indicate emphysema in this patient (D3). Presence of mucus is noted (D4). Corresponding H&E histology shows no signs of fibrosis but enlarged alveolar spaces compatible with emphysema (D5). ACF, airway centred fibrosis; ASM, airway smooth muscle; cHP, chronic hypersensitivity pneumonitis; DB, distal bronchiole; EA, enlarged alveolar spaces; EB-PS-OCT, endobronchial polarisation sensitive optical coherence tomography; EM, emphysema; F, fibrosis; IF, interstitial fibrosis; ILD, interstitial lung diseases; IPF, idiopathic pulmonary fibrosis; NSIP, non-specific interstitial pneumonia; PBM, peribronchial metaplasia; srILD, smoking-related ILD; TB, traction bronchiectasis.

Conventional EB-OCT enables identification of histological features of ILD.^16 23^ Additionally, EB-PS-OCT can distinguish fibrosis from other aetiologies of loss of aeration and provides information on fibrotic distribution. Representative EB-PS-OCT results acquired in the asthma controls and patients with ILD are shown in figures 3 and 4. Videos of EB-PS-OCT pullbacks corresponding to figures 3 and 4 are provided as online supplemental videos 1–5. Conventional EB-OCT intensity images (grey scale) have been overlapped with EB-PS-OCT optic axis orientation images (colour scale). In asthmatic controls, the added information of optic axis orientation images to EB-OCT intensity images confirmed the absence of fibrosis in the distal parenchymal area by the low amount of detected birefringence, although, as expected, in the proximal airway it revealed a large amount of birefringent airway smooth muscle as a sign of airway remodelling (figure 3, panel B1–B3). In fibrotic ILD cases, optic axis orientation images confirmed the presence of fibrotic tissue in the distal parenchymal area (figure 3, panel D1,2) by increasing amount of birefringence. Results show that in optic axis orientation images, fibrotic tissue fibres orientation varies significantly from region to region, as is indicated by the different colours present in fibrotic areas (figure 3, panel D1,D2). In contrast, non-pathological birefringent structures (eg, airway smooth muscle layers, perichondrium) are characterised by fibres highly organised in the same direction (figure 3, panel B1, B3, D1, D3).

10.1136/bmjresp-2023-001628.supp2 Supplementary video

10.1136/bmjresp-2023-001628.supp3 Supplementary video

10.1136/bmjresp-2023-001628.supp4 Supplementary video

10.1136/bmjresp-2023-001628.supp5 Supplementary video

10.1136/bmjresp-2023-001628.supp6 Supplementary video

Furthermore, EB-PS-OCT pullbacks acquired in vivo in patients with ILD identified fibrotic distribution and specific histological ILD hallmarks of different ILD subsets, which were visualised in the histology from the corresponding biopsies (figure 4).

In our cohort, all four IPF patients showed a subpleural distribution of fibrotic changes (figure 4A). For other, non-usual interstitial pneumonia patients, an airway centred (fibrotic cHP) or a more diffuse (fibrotic non-specific interstitial pneumonia) distribution of the fibrotic changes were noted (figure 4B,C). A patient with smoking-related abnormalities without fibrosis showed very little parenchymal birefringence, but mainly enlarged alveolar (EA) spaces with some mucus filling corresponding with emphysema (figure 4D). The ILD hallmarks visualised by EB-PS-OCT that were histologically confirmed included: dense destructive fibrosis, interstitial fibrosis and traction bronchiectasis in the IPF cohort. In this IPF cohort, no microscopic honeycombing was detected in EB-PS-OCT images, nor was it found in histology. In fibrotic cHP, we identified enlarged alveolar spaces among airway centred fibrosis. In one cHP patient, microscopic honeycombing was detected adjacent to the airway, indicating the airway centred rather than a subpleural oriented disease (online supplemental e-Figure 4). None of the above-mentioned hallmarks was visualised in any of the asthma controls. Thus, EB-PS-OCT identified characteristic features which were exclusively present in certain ILD subsets.

### Ex vivo PS-OCT results

**Figure 5:**
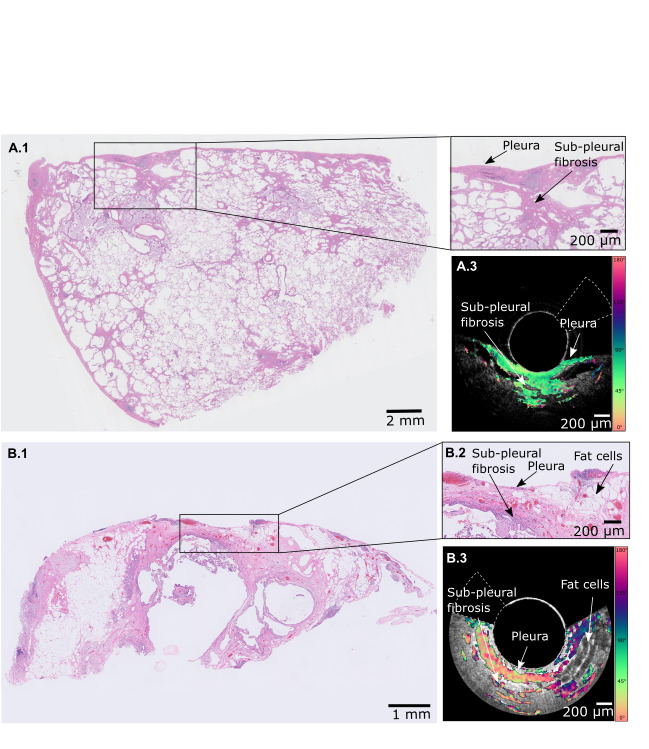
Matching histology of SLB and ex vivo EB-PS-OCT imaging. EB-PS-OCT imaging was performed pulling back the catheter along the surface of surgical lung biopsies from a patient with unclassified ILD (A) and a CHP patient (B). The specimens were cut perpendicular to the probe direction allowing for EB-PS-OCT and histology matching. In panel A) histology shows focal subpleural fibrosis (A1 and A2) and the cross-sectional image of the EB-PS-OCT shows birefringence in the same area, consistent with the presence of subpleural fibrosis (A3). In B, histology shows the presence of thickened pleura, subpleural fibrosis and fat cells (H&E stain B1 and B2). Corresponding to these structures, in the matching EB-PS-OCT cross-section are visualised birefringent areas and signal void cystic spaces (B3). Optic axis orientation images have been calibrated using the birefringent catheter sheath; this enabled to determine that birefringent structures in pink are circumferentially oriented, while birefringent structures in green are longitudinally oriented. The white dotted lines in OCT and PS-OCT images delineates the tissue area not optically accessible because of the presence of the wires feeding current to the motor. EB-PS-OCT, endobronchial polarisation sensitive optical coherence tomography; cHP, chronic hypersensitivity pneumonitis; ILD, interstitial lung diseases; OA, optic axis; SLB, surgical lung biopsy.

One-to one matches of fibrotic structures as visualised by PS-OCT and histological images were performed by ex vivo measurements on the resected SLB. Resulting PS-OCT ex vivo pullbacks were visually inspected and 21 confident matches between PS-OCT cross-sections and histology from 8 lung segments were found. The predominant features in these matches are regions of dense fibrotic tissue as visualised in histology that were identified by highly birefringent areas in PS-OCT cross-sectional images. PS-OCT images visualised superficial birefringent areas that were histologically confirmed as thickened pleura in the matching histology (figure 5A,B).

## Discussion

We found in vivo EB-PS-OCT to be a feasible, safe and minimally invasive imaging technique in patients with ILD. By using the fibrotic tissue specific-contrast provided by EB-PS-OCT, we demonstrated the ability of EB-PS-OCT to detect and quantify pulmonary birefringence. Results show differences in the mean birefringence area detected by EB-PS-OCT in the lung parenchyma from non-fibrotic cases (2.54%–6.75% range, in non-fibrotic ILD and asthma patients) and fibrotic cases (up to 21.01% in an IPF patient). The use of lung biopsies from corresponding lung segments confirmed that an increase in EB-PS-OCT detected birefringence corresponded to higher fibrosis extent in the biopsies. The ability of in vivo EB-PS-OCT to detect fibrosis is in line with a recent study by Nandy *et al*,^23^ in which quantitative birefringence measurements were compared across tissue types in patients with ILD. Unique to EB-PS-OCT in this study, is that besides the ability to detect birefringent structures, the orientation of fibres is identified in optic axis orientation images. This enables to distinguish different highly birefringent tissues such as airway smooth muscle and perichondrium from fibrotic parenchymal areas. Moreover, the identification of fibres orientation helps identifying the transition between distal and proximal airway and assists the distinction of specific ILD subsets based on the distribution of birefringence in the airways. We envision the use of EB-PS-OCT as an add-on diagnostic technique when HRCT is non-diagnostic or to evaluate whether fibrosis is present or progressive over time. To this end, we compared the ability of EB-PS-OCT to predict the extent of fibrosis in histology with HRCT. Although HRCT has a central role in ILD diagnosis, it has limitations in the distinction between inflammation and identification of fibrosis due to similar HRCT findings (ie, ground glass opacities) or very subtle radiological changes.^6 7 27–29^ Currently, the assessment of progressive pulmonary fibrosis relies on deterioration of clinical symptoms, pulmonary function tests or identification of progression on HRCT.^30^ This warrants a certain amount of irreversible damage before antifibrotic treatment can be initiated. Our findings show that EB-PS-OCT, with a resolution ~50 times higher than HRCT and with unique specific contrast for fibrosis, has better discriminative ability to evaluate the extent of fibrosis. Furthermore, in addition to conventional EB-OCT, by identifying birefringent areas EB-PS-OCT may distinguish areas of fibrosis from areas with a loss of aeration due to other factors such as atelectasis or inflammation.

In this study, ex vivo PS-OCT measurements on resected lung biopsies validated the potential of PS-OCT in identifying fibrotic tissue through one-to-one matches between PS-OCT cross-sections and histology. The presence of characteristic ILD features in EB-PS-OCT images was confirmed with corresponding histopathology. The absence of these hallmarks in EB-PS-OCT images from the asthma controls substantiates EB-PS-OCT ability in discriminating pathological features specific for ILD subsets.

This study has several limitations. Although our findings originate from a relatively high number of pullbacks, we recognise the number of patients included represents a limitation of this study and implicates the need for prospective validation in a larger patient cohort.

Additionally, the reported study population includes patients with ILD with an inconclusive clinicoradiological diagnosis, and therefore, extrapolation of the added value of EB-PS-OCT in cases with classifying HRCT pattern remains to be established.

Currently, no universally accepted method is available for the quantification of fibrosis in HRCT and in histopathology. The radiological and histological four-class fibrosis score of each segment was provided based on a pragmatic visual interpretation of the HRCT and lung biopsies rather than a validated scoring tool. Thus, a method which reflects daily practice of ILD-MDT sessions in which radiologists and pathologists evaluate the presence or progression of fibrosis based on their visual assessment was chosen for being feasible and reproducible as shown by the high weighted kappa score.

In the absence of healthy volunteers, we included three asthma patients who underwent bronchial thermoplasty as non-fibrotic controls. As bronchial thermoplasty was performed in the proximal airways, we do not expect the amount of birefringence in the distal lung parenchyma to be affected by it, which is line with normal HRCT and normal lung diffusion capacity. We recognise that asthma patients are not necessarily free from parenchymal fibrotic changes. However, normal diffusing capacity of the lungs for carbon monoxide and HRCT without parenchymal abnormalities suggest absence of fibrosis. In non-fibrotic patients (asthma and non-fibrotic ILD), detected birefringence ranged from 2.54% to 6.75%, meaning that a certain amount of birefringence should be considered as normal and reflects the normal architecture of the lung parenchyma and its connective tissue. Based on the current study, we cannot establish the optimal threshold to differentiate fibrosis from non-fibrotic areas, including normal alveolar tissue or areas with an increased birefringent signal due to other reasons than fibrosis (ie, atelectasis). More research is needed to establish such a threshold.

As a reference standard for EB-PS-OCT detected fibrosis and ILD hallmarks, we included TBCB besides SLB as histological reference, which considering its limitation in size may increase the risk of sampling error. However, a growing body of evidence shows adequate diagnostic performance of TBCB as is illustrated by the recent guideline recommendations for the use of TBCB as a first step diagnostic approach for tissue diagnosis.^30–33^ Our study adds to the hypothesis that EB-PS-OCT can potentially prevent invasive tissue acquisition by providing ‘optical’ histological ILD features and extent of fibrosis without the need for tissue extraction. Furthermore, due to its minimally invasive character, longitudinal measurements may identify presence and/or progression at an early stage or when HRCT is inconclusive.

In conclusion, EB-PS-OCT is feasible and safe and enables accurate in vivo quantification of fibrosis and identification of ILD features/hallmarks in the lung parenchyma of patients with ILD in a minimally invasive way. On further validation, this novel imaging technique has potential to influence ILD diagnostic, therapeutic and monitoring strategies omitting the need for tissue acquisition with added value to the currently widely used and accepted HRCT.

## Data Availability

Data are available on reasonable request. Data are available by sending a request to j.f.de.boer@vu.nl.
