# Supplementary material for: In vivo polarisation sensitive optical coherence tomography for fibrosis assessment in interstitial lung disease: a prospective, exploratory, observational study
Source: BMJ Open Respir Res. 2023 Aug 8;10(1):e001628. doi: 10.1136/bmjresp-2023-001628 (PMC10414088; doi:10.1136/bmjresp-2023-001628)
Supplement: Supplementary data [file bmjresp-2023-001628supp001.pdf]

Supplementary

e-Table 1

**Inclusion criteria**
• >18 years of age<br>• ILD on HR-CT<br>• No classifying diagnosis based on clinical data including laboratory results and HR-CT<br>• ILD-MDT panel indication for histology by lung biopsy
**Exclusion criteria**
• Inability and willingness to provide informed consent<br>• Inability to comply with study protocol<br>• Prothrombin time international normalized ratio (INR) >1.3<br>• Use of clopidogrel, or other new anti-platelet therapy, or anticoagulant drugs that cannot be stopped temporarily<br>• Thrombocytopenia <70x10^9/L<br>• History of pulmonary hypertension (systolic pulmonary artery pressure > 50 mmHg)<br>• Diffusing capacity < 30%<br>• Forced vital capacity <50%<br>• Forced expiratory volume in the first second (FEV1) <0,8L or <50% of predicted value<br>• Body mass index >35<br>• Pregnancy

e-Table 1 In- and exclusion criteria for study participation.

e-Appendix 1:
EB-PS-OCT data processing and fibrosis quantification:

Analysis of the acquired EB-PS-OCT raw-data was performed in MATLAB R2021a (MathWorks) in post-processing. Conventional OCT intensity images were generated from raw data and displayed in grayscale with 55 dB dynamic range. For all the acquired frames, after compensating for the polarization mode dispersion induced by the optical fibers in the system [1], birefringence properties of tissue were extracted using the differential Mueller matrix algorithm developed by Villiger et al [2]. To retrieve depth-resolved optic axis orientation images, the presence of preceding birefringent layers of tissue with a different optic axis was compensated [3]. Moreover, the variation of the incident polarization state induced by the rotation of the motor at the tip of the catheter was corrected. Absolute orientation of the tissue optic axis was extracted using the catheter sheath, made of homogenously oriented birefringent material, as calibration tool [4]. This enabled the discrimination of parallel and circumferentially oriented birefringent tissue structures. Birefringent structures were automatically segmented by thresholding optic axis orientation images based on optic axis uniformity over a small region (threshold value of 0.55) [5]. The optic axis uniformity was

calculated over a two-dimensional spatial kernel (4x18 pixels), which correspond to 26 µm in depth and 7° along the azimuthal direction. In each frame the percentage of birefringent area over the total OCT cross section was calculated, extending from the outer sheath of the catheter to an imaging depth of 790 µm. To quantify fibrosis in the lung parenchyma, a distinction between peripheral parenchymal (distal) area of the pullback imaging the alveolar compartment and proximal part of each pullback imaging the airways was made. The peripheral parenchymal area of the pullback (100-350 adjacent frames per pullback) was identified in post-processing by i) the presence of alveoli adjacent to the EB-PS-OCT probe and ii) the absence of birefringent airway smooth muscle. This distinction was performed by a reader with expertise in EB-PS-OCT who manually assessed individual cross-sectional images based on the above criteria.

e-Appendix 2:

Criteria for the categorical fibrosis scoring system for histology:

Fibrosis score 0:<br>No fibrosis	Fibrosis score 1:<br>Mild fibrosis	Fibrosis score 2:<br>Moderate fibrosis	Fibrosis score 3:<br>Severe fibrosis
Normal lung parenchyma without presence of fibrosis	Focal slight increase of alveolar wall thickness by collagen	Moderate, focal, increase in alveolar wall thickness by collagen	Diffuse prominent increase in thickness of alveolar walls by collagen

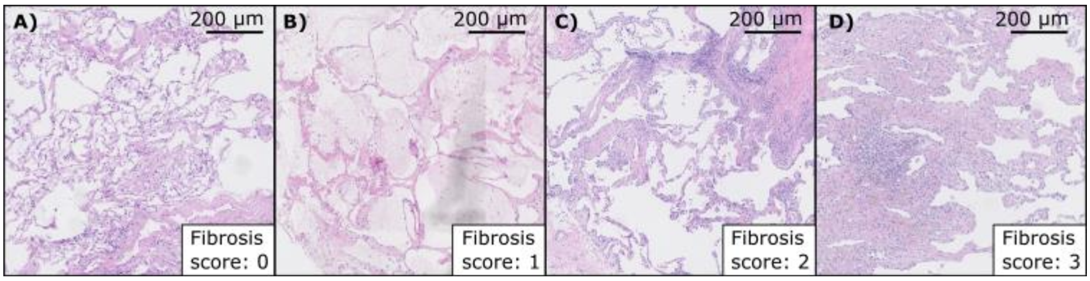

Hematoxylin-eosin (H&E) stained histologic sections for different fibrosis scores.
A) fibrosis score 0= no fibrosis. The image shows prominent collapse of thin alveolar walls, B) fibrosis score 1= mild fibrosis, C) fibrosis score 2= moderate fibrosis, C) fibrosis score 3= severe fibrosis)

Criteria for the categorical fibrosis scoring system for HRCT:

Fibrosis score 0: No fibrosis	Fibrosis score 1: Mild fibrosis	Fibrosis score 2: Moderate fibrosis	Fibrosis score 3: Severe fibrosis
lung parenchyma without reticulation or anatomical distortion	some reticulation with very little to none architectural distortion	reticulation with clear but limited signs of distortion and traction bronchiectasis	Reticulation with extensive distortion and traction bronchiectasis

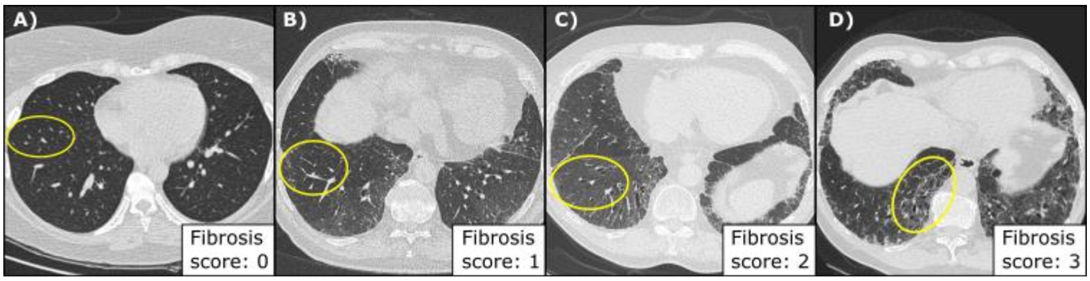

High resolution computed tomography (HRCT) scans for different fibrosis scores.
A) fibrosis score 0= no fibrosis, B) fibrosis score 1= mild fibrosis, C) fibrosis score 2= moderate fibrosis, C) fibrosis score 3= severe fibrosis). For each panel the yellow circle in the HRCT section indicates the segment for which the fibrosis score is assessed.

Figures

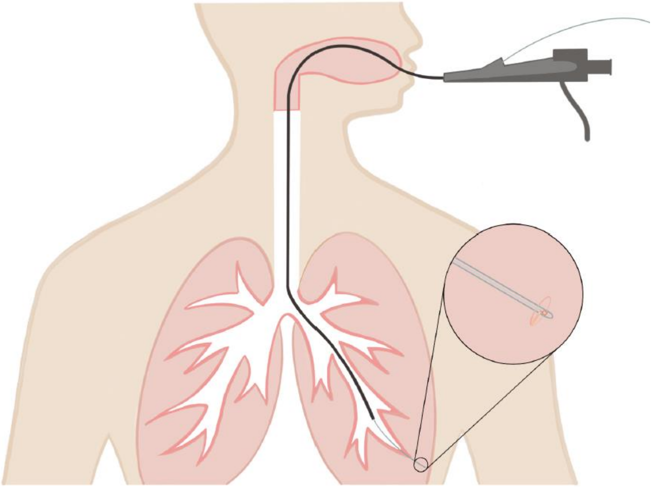

e-Figure 1. Schematic drawing of the *in-vivo* endobronchial polarization sensitive optical coherence tomography (EB-PS-OCT) procedure. Volumetric EB-PS-OCT images were acquired by advancing the EB-PS-OCT probe through the working channel of the bronchoscope to the periphery of each lung segment until pleural resistance.

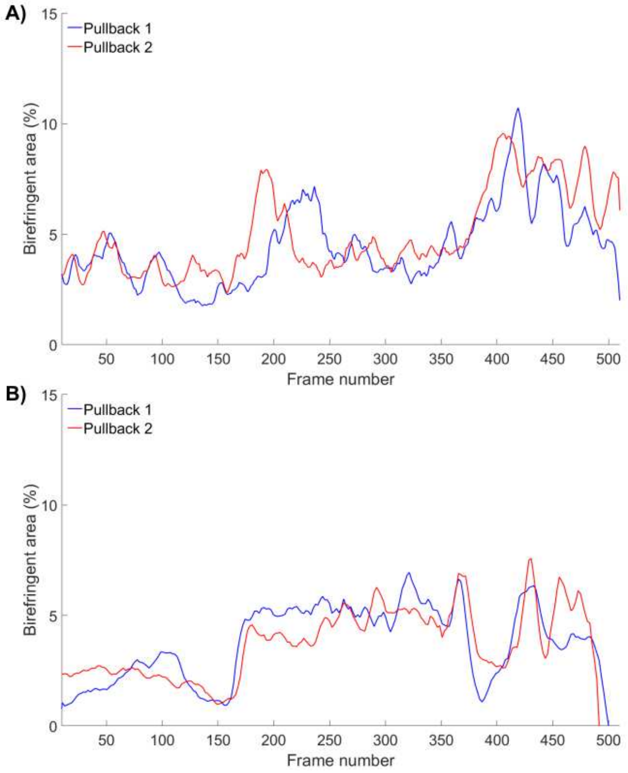

e-Figure 2. Reproducibility of the birefringent content detected by *in-vivo* endobronchial polarization sensitive optical coherence tomography (EB-PS-OCT).

A) EB-PS-OCT detected birefringent content in two consecutive pullbacks (pullback 1 in blue and pullback 2 in red) acquired in segment RB10 from a cHP patient.

B) EB-PS-OCT detected birefringent content in two consecutive pullbacks (pullback 1 in blue and pullback 2 in red) acquired in segment RB9 from a different cHP patient.

EB-PS-OCT detected birefringent content is expressed as percentage of birefringence over the total PS-OCT cross-section extending from the catheter sheath.

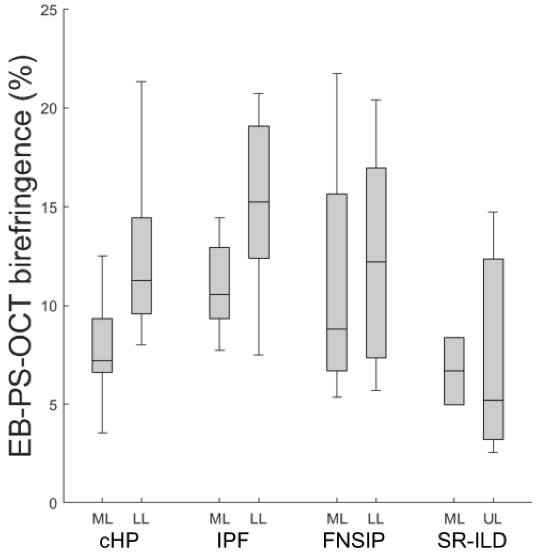

e-Figure 3 In ILD patients with inconclusive or inconsistent HRCTs, EB-PS-OCT detected fibrosis in the alveolar compartment of the lower lobe (LL), resulted to be consistently higher than in the alveolar compartment of the middle lobe (ML) in patients diagnosed with IPF and FNSIP. In patients diagnosed with cHP, EB-PS-OCT detected fibrosis in the alveolar compartment of the lower lobe (LL), resulted to be significantly higher than in the alveolar compartment of the middle lobe (ML) (p value=0.069). In SR-ILD the EB-PS-OCT detected fibrosis in the alveolar compartment of the middle lobe resulted to be higher than in the alveolar compartment of the upper lobe (UL).

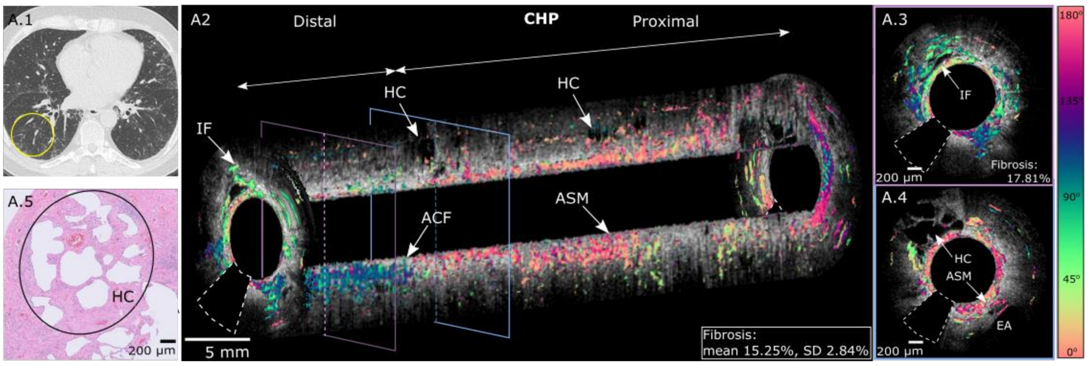

e-Figure 4 High resolution computed tomography (HRCT), Hematoxylin-eosin (H&E) stained histologic section images and PS-OCT images of lung segment RB9 from a patient diagnosed with chronic hypersensitivity pneumonitis (cHP). The PS-OCT pullback illustrates presence of airway centered fibrosis (ACF) around the airways proximal to regions with preserved alveolar architecture.

A.1) HRCT image, the yellow circle indicates the region of interest (segment RB9) in which the biopsy from figure **A.5** and PS-OCT images from figure **A.2-4** were acquired. **A.2**) Volumetric PS-OCT pullback shows the presence of airway centred fibrosis (ACF). **A.3,4**) OCT and OA cross

sectional images, taken from the locations of the PS-OCT pullback **(A.2)** show presence of interstitial fibrosis (IF), microscopic honeycombing (HC) and airway smooth muscle (ASM). **A.5**) Hematoxylin-eosin (H&E) stained histologic section from transbronchial cryobiopsy of corresponding lung segment confirmed presence of microscopic honeycombing (marked with a black circle).

The white dotted lines in PS-OCT images delineate the tissue area not optically accessible because of the presence of the wires feeding current to the motor.

Extended Data References:

References:

[1] Braaf, B., et al. (2014). "Fiber-based polarization-sensitive OCT of the human retina with correction of system polarization distortions." Biomed Opt Express **5**(8): 2736-2758.

[2] Villiger, M., et al. (2016). "Deep tissue volume imaging of birefringence through fibre-optic needle probes for the delineation of breast tumour." Sci Rep **6**: 28771.

[3] Willemse, J., et al. (2020). "In Vivo 3D Determination of Peripapillary Scleral and Retinal Layer Architecture Using Polarization-Sensitive Optical Coherence Tomography." Transl Vis Sci Technol **9**(11): 21.

[4] Villiger, M., et al. (2018). "Optic axis mapping with catheter-based polarization-sensitive optical coherence tomography." Optica **5**(10): 1329-1337.

[5] Feroldi, F., et al. (2019). "In vivo multifunctional optical coherence tomography at the periphery of the lungs." Biomed Opt Express **10**(6): 3070-3091.
